# Pharmacological treatment of hypertriglyceridemia-induced acute pancreatitis during pregnancy: A case report and literature review

**DOI:** 10.1097/MD.0000000000041810

**Published:** 2025-03-14

**Authors:** Pengchuan Liu, Pengfei Zhao, Ting Zhao, Lihong Yu

**Affiliations:** a Department of Clinical Pharmacy, Weifang People’s Hospital, Weifang, Shandong Province, China.

**Keywords:** acute pancreatitis, ezetimibe, fenofibrate, octreotide, ulinastatin

## Abstract

**Rationale:**

Hypertriglyceridemia-induced acute pancreatitis during pregnancy is a rare and severe condition that poses significant risks to both maternal and neonatal health; however, there is a lack of standardized treatment guidelines and restrictions on therapeutic options during pregnancy.

**Patient concerns:**

We present a case of acute pancreatitis caused by gestational hypertriglyceridemia and conduct a literature review regarding the safety of the primary therapeutic drugs used during pregnancy.

**Diagnoses:**

A 32-year-old female patient, who was 24 weeks pregnant, experienced postprandial abdominal pain accompanied by nausea and vomiting. A computed tomography scan confirmed the diagnosis of acute pancreatitis, and her serum triglyceride levels were found to be 57.00 mmol/L.

**Interventions:**

Upon admission to the hospital, the patient’s treatment encompassed intravenous hydration, blood filtration, and the administration of octreotide and ulinastatin to suppress the pancreatic secretion of fluid and enzymes. In addition, she underwent lipid-lowering therapy with fenofibrate and ezetimibe.

**Outcomes:**

The patient recovered and was discharged, with no recurrence of pancreatitis throughout her pregnancy. At 36 + 1 weeks of gestation, the patient underwent a cesarean section due to premature rupture of membranes, inevitable preterm birth, and fetal position right sacrum anterior, delivering a healthy female newborn.

**Lessons:**

The safety evidence for acute pancreatitis treatment drugs such as octreotide, ulinastatin, and lipid-lowering medications during pregnancy is not fully sufficient. However, considering the severity of the disease, which often occurs in the second and third trimesters of pregnancy, cautious use may be warranted when the benefits outweigh the risks.

## 1. Introduction

Hypertriglyceridemia-induced acute pancreatitis during pregnancy, while rare, poses a significant threat to maternal and neonatal health. Owing to limited treatment options during pregnancy, the absence of consensus treatment guidelines presents a significant challenge for healthcare providers. This article discusses the treatment and maternal-infant outcomes of a case of hypertriglyceridemia-induced acute pancreatitis during pregnancy, conducts a critical review of the safety profiles of pharmacological treatments, and provides clinical guidance for the pharmacological management of such cases.

## 2. Case

A 32-year-old pregnant woman at 24 weeks of gestation, gravida 2 para 0, presented to the emergency department with abdominal pain lasting 9 hours. The patient developed generalized abdominal pain after eating 9 hours prior, characterized by continuous distension and pain, accompanied by nausea and vomiting of gastric contents 3 times. The symptoms persisted without relief, prompting her visit to our hospital where she was admitted to the intensive care unit. Her body temperature was 36.4°C, heart rate was 72 beats/minute, respiratory rate was 16 breaths/minute, and blood pressure was 114/62 mm Hg. Obstetric examination revealed a gravid uterus, normal fetal heart rate, and no uterine contractions. Laboratory tests revealed the following results: extremely elevated triglyceride (TG): 57.00 mmol/L (normal range 0.40–2.25 mmol/L), total cholesterol (TC): 20.7 mmol/L (2.9–5.69 mmol/L), low-density lipoprotein cholesterol (LDL-C): 5.93 mmol/L (1.5–4.3 mmol/L), high-density lipoprotein cholesterol (HDL-C): 2.74 mmol/L (1.0–2.1 mmol/L), serum glucose: 7.6 mmol/L (3.9–6.1 mmol/L), amylase: 88 U/L (35–135 U/L), white blood cell count: 15.71 × 10^9^/L (3.5–9.5 × 10^9^/L), C-reactive protein (CRP): 12.0 mg/L (0–8 mg/L), elevated urinary amylase: 1180 U/L (0–440 U/L). Additionally, the blood samples were chylous. Abdominal ultrasonography suggested fatty liver, whereas computed tomography of the abdomen and pelvis indicated acute pancreatitis.

**Figure 1.**
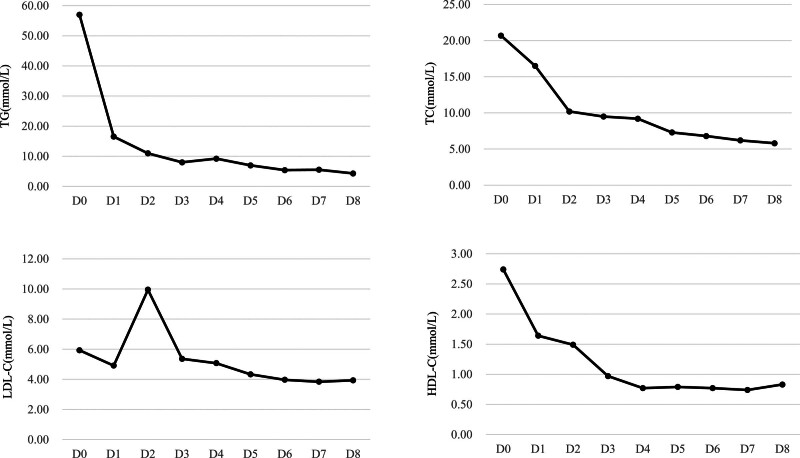
Trend of blood lipids during hospitalization. HDL-C = high-density lipoprotein cholesterol, LDL-C = low-density lipoprotein cholesterol, TC = total cholesterol, TG = triglyceride.

**Figure 2.**
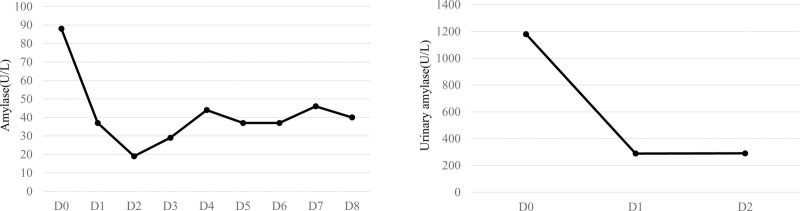
Trend of Amylase level during hospitalization.

Following admission, the patient was provided supportive care, including fasting and fluid resuscitation. Continuous veno-venous hemofiltration was performed to reduce blood lipid levels. Pharmacological treatment included inhibitors of gastric acid and pancreatic enzyme secretion (esomeprazole, octreotide, and ulinastatin), and an anti-infective agent (meropenem). On day 4, owing to blood viscosity and high abdominal pressure leading to poor blood flow in the tubing, hemofiltration could not be completed. Lab tests then showed TC at 9.2 mmol/L, TG at 9.21 mmol/L, LDL-C at 5.07 mmol/L, HDL-C at 0.77 mmol/L, amylase at 44 U/L, and CRP at 173.9 mg/L. Upon obtaining informed consent from the patient, fenofibrate and ezetimibe were prescribed for lipid management. On day 6, the patient’s abdominal pain was significantly alleviated, and laboratory tests showing TC at 6.8 mmol/L, TG at 5.39 mmol/L, LDL at 3.97 mmol/L, HDL at 0.77 mmol/L, amylase at 37 U/L, and CRP at 34.4 mg/L. The patient was discharged on the ninth day in good condition, advised to follow regular prenatal checkups and a fat-restricted diet, continue taking fenofibrate, and monitor blood lipid levels. Changes in blood lipids from hospitalization to discharge are shown in Figure 1, and amylase levels are shown in Figure 2. At 31 weeks of gestation, blood lipid levels were reevaluated with TC at 7.9 mmol/L, TG at 16.22 mmol/L, LDL-C at 2.53 mmol/L, HDL-C at 1.01 mmol/L, and amylase at 33U/L. The patient reported nonadherence to medication after discharge from the hospital, prompting a repeat prescription of fenofibrate. At 36 + 1 weeks of gestation, due to premature rupture of membranes, inevitable preterm birth, and fetal position RSA, cesarean section was performed. A healthy female neonate weighing 3700 g was delivered with Apgar scores of 10 and 10 at 1 and 5 minutes, respectively.

## 3. Discussion

Acute pancreatitis in pregnancy is a rare and serious complication of gestation, potentially leading to adverse maternal and fetal outcomes, with an incidence rate of approximately 0.225 to 2.237 per 1000 pregnancies. Cholelithiasis emerged as the principal cause, with hypertriglyceridemia being the second leading etiology. Notably, studies from Eastern regions, especially China, indicate a greater incidence of hypertriglyceridemia-related cases in comparison to Western data.^[1]^ Over half of the cases of acute pancreatitis during pregnancy are mild, while about one-third escalate to severe pancreatitis. Despite improvements in maternal and fetal outcomes due to advancements in diagnosis and treatment-reducing maternal mortality from 3.3% in 2010 to 2015 to 2.4% in 2016 to 2020 and fetal mortality from 13.0% to 12.6%,^[2]^ there are currently no standardized treatment guidelines for acute pancreatitis in pregnancy. The limited options for medication during pregnancy and absence of standardized treatment guidelines present significant challenges for clinical management.

For hypertriglyceridemia-induced acute pancreatitis during pregnancy, in addition to the general treatment for acute pancreatitis, lipid-lowering therapy is required to rapidly decrease triglyceride levels and aim to maintain them below 5.65 mmol/L.^[3]^ Conservative management includes fluid resuscitation, acid suppression, inhibition of pancreatic fluid and enzyme secretion, lipid-lowering treatment, nutritional support, use of antimicrobial drugs, and pain management. Pregnancy safety data for related therapeutic drugs primarily come from case reports and small-scale studies. Given the recent publication of new case reports and systematic reviews, this study combined the latest literature to discuss the pregnancy safety of key medications, namely octreotide, ulinastatin, fenofibrate, and ezetimibe, as follows.

Octreotide, widely utilized for acute pancreatitis treatment owing to its ability to inhibit pancreatic enzyme secretion, is capable of crossing the placental barrier. Nevertheless, data on its safety during pregnancy are limited and are primarily derived from isolated case reports and small-scale case series. In most reports, the use of octreotide during pregnancy has not been associated with adverse pregnancy outcomes, with only isolated cases reporting fetal growth restriction^[4,5]^ and neonatal necrotizing enterocolitis.^[6]^

Ulinastatin, a protease inhibitor extracted from human urine, broadly inhibits the activity and release of enzymes (such as trypsin) associated with the progression of pancreatitis. It also stabilizes lysosomal membranes, reduces the release of inflammatory mediators, improves pancreatic circulation, and decreases the occurrence of complications, and is commonly used in the clinical treatment of acute pancreatitis. Analysis of ulinastatin concentration in the urine of different populations indicated that the concentration in fetal urine is relatively high, nearly 50 times that in adult urine. Animal experiments have demonstrated the distribution of ulinastatin in the placenta and milk. Ulinastatin vaginal suppositories are used in Japan to prevent preterm labor, with no reported adverse pregnancy outcomes attributable to the drug itself.^[7,8]^ Hara et al^[9]^ reported a case of a 36-year-old woman at 11 weeks of gestation, who received intravenous ulinastatin during hospitalization for acute pancreatitis, and underwent cesarean section due to placenta previa at 37 + 2 weeks, with an infant weighing 2694 g at birth, showing normal physical examination and clinical manifestations. Given these observations, while comprehensive safety data on intravenous ulinastatin use during pregnancy remain incomplete, it is generally regarded as relatively safe.

Fenofibrate is a prodrug that is rapidly hydrolyzed by esterases to its active metabolite, fenofibric acid, after oral administration. It is capable of reducing triglyceride levels by 40% to 50%, making it the preferred drug for treating hypertriglyceridemia. The elimination half-life of fenofibric acid is approximately 20 hours. It is currently unclear whether fenofibrate or fenofibric acid crosses the human placenta, but their high plasma protein-binding rate (approximately 99%) may limit placental transport. Animal studies have not shown evidence of embryo-fetal toxicity at the recommended doses, although higher doses have resulted in adverse outcomes. Human experience in using fenofibrate during pregnancy is limited, primarily derived from a small number of case reports. Previous reports have not found an association between fenofibrate exposure during pregnancy and congenital anomalies in the fetus; however, most case reports involve medication use during the second and third trimesters,^[10–12]^ with only 1 case reported in the first trimester.^[13]^ This may be related to the increased incidence of hyperlipidemic pancreatitis in the second and third trimesters.

Ezetimibe is rapidly absorbed after oral administration and extensively metabolized in the intestine and liver. Both the parent compound and its metabolites possess pharmacological activity and exhibit a high degree of plasma protein binding (>90%) with an elimination half-life of approximately 22 hours. It is currently unclear whether ezetimibe and its active metabolites cross the human placenta, but their high plasma protein-binding rate may limit placental transport. Animal studies have suggested that ezetimibe poses a low risk to embryos and fetuses, with an increased incidence of skeletal abnormalities only found at extremely high doses. To date, there have been 2 reports of ezetimibe use during human pregnancy, 1 in the first trimester^[14]^ and another in the second trimester,^[11]^ with no adverse outcomes observed. Considering the sparse data on ezetimibe use in pregnant women, accurately evaluating its embryonic and fetal safety profiles presents a challenge.

Statins have traditionally been contraindicated in pregnant women because of their potential to inhibit cholesterol synthesis, which is necessary for fetal growth and development, with animal studies showing teratogenicity and human pregnancy exposure linked to fetal malformations in early reports. However, over the past 30 years, experiences and studies involving the accidental use of statins before and during pregnancy have not shown an association with congenital malformations or other adverse pregnancy outcomes. Consequently, in July 2021, the United States Food and Drug Administration (FDA) required the removal of contraindications for statin use during pregnancy. Although the FDA still does not recommend the routine use of statins during pregnancy, its use is now permitted in women with familial hypercholesterolemia, atherosclerotic cardiovascular disease, or severely elevated LDL-C levels when the benefits outweigh the risks.^[15]^

Moreover, the use of Omega-3 fatty acids to reduce triglycerides in pregnant women is relatively safe; however, prescription-strength products are not available in China. Insulin enhances lipoprotein lipase activity, leading to chylomicron degradation, and heparin stimulates the release of lipoprotein lipase, thereby reducing serum TG levels. However, the use of insulin in non-diabetic patients carries a risk of hypoglycemia, and the long-term use of heparin may lead to overconsumption of lipoprotein lipase.^[3]^

Hypertriglyceridemia-induced acute pancreatitis during pregnancy is a rare and serious complication, with no established clinical practice guidelines available to date, and current recommendations are primarily derived from observational data in the form of case reports. The safety evidence for acute pancreatitis treatment drugs such as octreotide, ulinastatin, and lipid-lowering medications during pregnancy is not fully sufficient. However, considering the severity of the disease, which often occurs in the second and third trimesters of pregnancy, cautious use may be warranted when the benefits outweigh the risks.

## Acknowledgments

The authors would like to thank Jihong Zhang of Weifang People’s Hospital for her consultation invitation.

## Author contributions

**Resources:** Pengchuan Liu, Pengfei Zhao, Ting Zhao.

**Writing – original draft:** Pengchuan Liu.

**Writing – review & editing:** Lihong Yu.
